# Promoting Oral Health for Older Adults in the Asia-Pacific Region

**DOI:** 10.1016/j.identj.2025.100886

**Published:** 2025-09-22

**Authors:** Alice Kit Ying Chan, Chun-Pin Lin, Olabode Ijarogbe, Elham Kateeb, Hiroshi Ogawa, Syed Mahmood Shah, Fernando Fernandez, Ting-Chen Chen, Ting-Yi Renn, Chun Hung Chu, Wei-Jen Chang

**Affiliations:** a Faculty of Dentistry, The University of Hong Kong, Hong Kong, China; b Department of Dentistry, National Taiwan University Hospital, Taipei, Taiwan; c Graduate Institute of Clinical Dentistry, School of Dentistry, National Taiwan University, Taipei, Taiwan; d Asia Pacific Dental Federation, Manilla, Philippine; e Department of Restorative Dentistry, Faculty of Dental Sciences, College of Medicine, University of Lagos, Lagos, Nigeria; f Oral Health Research and Promotion Unit, Al-Quds University, Jerusalem, State of Palestine; g Division of Preventive Dentistry, Department of Oral Health Science, Graduate School of Medical and Dental Sciences, Niigata University, Niigata, Japan; h Department of Orthodontics, Muhammad Dental College, Mirpurkhas, Sindh, Pakistan; i Department of Oral Health, Ministry of Health and Welfare, Taipei City, Taiwan; j School of Dentistry, College of Oral Medicine, Taipei Medical University, Taipei, Taiwan; k Dental Department, Shuang-Ho Hospital, Taipei Medical University, New Taipei City, Taiwan

**Keywords:** Older adults, Asia-Pacific, Periodontal disease, Caries

## Abstract

The population of the Asia-Pacific region is ageing, and oral diseases are prevalent. Most Asian older adults had periodontal disease, and two-thirds of them had untreated caries. Ageing, medical conditions, and polypharmacy make older adults vulnerable to oral diseases. Oral health is related to systemic health, morbidity, and mortality, making it a key element in healthy ageing for older adults. Recognizing the importance of oral health in the general well-being of older adults, the Asia Pacific Dental Federation hosted the Asia Pacific Dental Congress 2024 and convened public health experts to develop recommendations for promoting oral health in older adults across Asia-Pacific countries and regions. The experts reviewed the current literature and recommended that stakeholders share roles in promoting oral health for older adults. Policymakers should implement universal coverage for oral health care and integrate oral health into general health care for older adults. Professional organizations and dental associations should conduct interprofessional training in geriatric oral care, perform surveillance of oral diseases, organize community-based oral health education, and implement programs for oral disease prevention for older adults. Academics should include geriatric oral care in the curricula for dental schools and other health-related schools. They should also conduct research to develop evidence-based best practices for older adults. Dental professionals should perform regular examinations to detect oral diseases early and use the common risk factors approach to promote oral and general health in older adults. Additionally, dental professionals should provide preventive measures and adopt minimally invasive approaches to managing oral diseases.

## Introduction

The population is ageing. The older adult population is growing in number and proportion worldwide due to increased life expectancy and a low fertility rate.^1^ United Nations reported that the number of older adults aged 65 or above will double from 761 million in 2021 to 1.6 billion in 2050 with 1 in 6 among the world population.^1^ More than 60% of the global increase in older adult population will come from Asia-Pacific region and the demographic transition of the world’s oldest countries will shift from Europe to Eastern and South-Eastern Asia with 1 in 3 of the population aged 65 or above in Japan, Hong Kong, Republic of Korea, Singapore and Taiwan in 2050.^1^^,^^2^

Living longer does not always come with good health. As people age, they are prone to have a decline in their cognitive, physical and medical conditions.^3^ Globally, almost half (46%) of the older adults have disabilities and most of them live in Asia-Pacific region.^4^ Age changes, medical conditions and polypharmacy make older adults susceptible to oral diseases.^5^ However, older adults may not easily receive regular oral health care because of their ill health, physical disability or functional decline. Moreover, expensive dental treatment fees without any public funding or insurance coverage further discourage them from seeking oral health care. Therefore, the prevalence of oral diseases in older adults still remains high.

Dental caries, periodontal diseases, tooth loss, hyposalivation, and oral cancer are the most common oral diseases of older adults.^3^ Half of the global older adult population had untreated dental caries, and two-thirds of them suffered from periodontal diseases.^6^^,^^7^ However, the situation is even worse in Asian adults. Two-thirds of the older adults had their caries left unattended.^6^ Almost all Chinese older adults had periodontal diseases and one in seven of them suffered from severe periodontitis.^8^ Tooth loss is the end point of untreated oral diseases and the risk of tooth loss increased with ages mainly affecting older adult groups.^9^ The risk of tooth loss has been reduced in the past decades due to the improvement in medical standard and socioeconomic conditions.^9^ However, there is an abrupt increased risk of severe tooth loss in old adults aged 75 or above who are likely to be dependent and institutionalized.^9^ In Australia, one in five of older adults aged 75 or above were edentulous.^10^ Tooth loss, hyposalivation and oral cancer may lead to oral function decline in older adults.^11^ Two in three of Japanese community-dwelling older adults were diagnosed with oral hypofunction.^12^

Oral health is linked to systemic health.^13^ Numerous studies demonstrated that oral health was associated with several medical conditions such as cardiovascular diseases, dementia and diabetes through their shared inflammatory pathways.^14^ Tooth-loss impacts chewing and swallowing function while reducing nutritional intake in older adults.^15^ It also negatively affects aesthetics, communication, social interaction and oral-related quality of life in older adults.^3^ Oral function decline has recently received attention as an important oral problem in older adults because it was associated with general functional capacity, frailty and disability in older adults.^11^ Oral health has been affirmed by the World Health Organization (WHO) and FDI World Dental Federation as the key element of healthy ageing in older adults.^16^^,^^17^ Improving oral health will be the paramount public health issue for the ageing population in the coming decade.

Oral health care has traditionally operated separately from the mainstream health care system.^18^ Oral health is thus neglected in health care policy; least alone having any oral health care policy specific for older adults.^19^ Oral health care services in most Asia-Pacific region such as India, Hong Kong, and Australia are mainly provided by private dental sectors and is paid out of the pockets by their citizens.^5^^,^^19^^,^^20^ Older adults with special needs or at low socioeconomical status may be eligible for public or funded dental services in some countries. For example, older adults with low income are eligible for public dental services with a copayment in Australia whereas those who fulfil the financial eligibility criteria or are institutionalized can receive funded dental treatment in Hong Kong.^5^^,^^19^ Oral health care in some countries, like China, Thailand and Japan, has been included in the universal health coverage.^19^ However, the range of dental treatment and the proportion of the dental treatment fee covered varied among countries. Basic dental treatment including preventive care, restorations and root canal treatment is covered in Thailand.^21^ Nevertheless, older adults in Thailand need to pay full fee for any rehabilitation work such as crowns, dentures and dental implants.^21^ Japan covered a wide range of dental treatment including prosthodontic treatment and older adults pay at a reduced rate of 10% to 20% of the cost only.^22^

Most countries do not have any specific oral health care coverage for older adults regarding their increased dental needs and barriers to dental access. Thailand and Japan are the only two Asia-Pacific countries integrated oral health care into their general health care services and implemented several oral health policies specific for older adults to improve their oral health and oral function.^11^^,^^21^ Currently, most Asia-Pacific region is still facing various challenges in implementing oral health policies to improve oral health in older adults.^19^ These challenges include shortage of dental personnel, limited resources in remote areas, lack of expertise in geriatric dentistry, lack of budget, inadequate training of geriatric oral care for healthcare providers and insufficient collaboration between oral and general health care professionals.^19^

The aims of the policy statement are to highlight the importance of oral health in older adults and to provide recommendations for different stakeholders to promote oral health for older adults in the Asia-Pacific region.

## Methods

The Asia Pacific Dental Federation acknowledged the importance of oral health in older adults and organized a workshop on promoting oral health care in older adults during the Asia Pacific Dental Congress 2024. The workshop aimed to develop recommendations for different stakeholders to promote oral health care for older adults in the Asia-Pacific region. A working group was hence formed by a panel of health experts in geriatric oral care. They reviewed the current literature on oral health care for older adults to identify the challenges in implementing oral health care for older adults and formulated recommendations for different stakeholders to promote oral health for older adults in the Asia-Pacific region. The working group convened on 2nd May 2024 at the 45th Asia Pacific Dental Congress in Taipei, Taiwan, to discuss and consent the recommendations.

## Recommendations for policymakers

### To include oral health in universal health coverage

Oral health inequalities are the main reason for poor oral health in older adults. Older adults with low educational level, underprivileged socioeconomic status and disabilities are at risk of poor oral health.^13^ Universal health coverage aims to reduce health inequalities by ensuring all people receive all range of health services from promotive and preventive care to curative, rehabilitative and palliative intervention that they needed of sufficient quality regardless of their financial status.^23^ The WHO and the FDI World Dental Federation have proposed to include oral health in universal health coverage to make oral health care accessible, available and affordable to all including older adults with no one left behind.^16^^,^^24^

Universal oral health coverage should be the goal, but is unlikely to be achieved in one step.^24^ It requires substantial resources, manpower, and budget. There will be country- and region-specific challenges in implementation.^24^ And the depth and range of treatment covered depends on the maturity of the health care system.^25^ Generally, the health care system should have an efficient and experienced governing system, a resilience, affordable and equitable financing structure, access to essential medicines and technologies and a sufficiently trained and collaborated health workforce.^25^ Japan has implemented universal oral health coverage and the percentage of older adults aged 80 with 20 natural teeth or more has increased from 7% in 1989 to 51% in 2016.^24^

### To integrate oral health into general health care system

Oral health care traditionally has been operated as an isolated field from the mainstream of medicine. However, emerging evidence has demonstrated that oral and systemic health are interrelated with some shared common risk factors.^13^^,^^26^^,^^27^ Integrating oral health into general health care services in older adults can improve their oral and systemic health simultaneously and synergistically through common risk factor approach.^18^^,^^28^ A patient-centred, integrated and multidisciplinary management plan within and among different levels of health care professionals will improve older adults’ overall health outcomes.^29^ Moreover, the integration of oral and general health care can facilitate early detection of systemic diseases, reinforce health surveillance, aid data sharing, allocate resources and workforce more efficiently within the healthcare system.^18^

The WHO has advocated the integration of oral health into general health care since 2008, but the integration is still at the earliest stage.^30^ Compartmentalised health care system, difficulties in sharing information, uncoordinated referral system, poor interprofessional communication, shortage of workforce and lack of interprofessional training are the barriers for the integration.^31^^,^^32^ Inclusion of oral health into national health care system is the fundamental step for the integration. Various strategies like setting up multidisciplinary clinics, delivering geriatric oral health care training to all allied health care workers, building an interdisciplinary network for data sharing, and having referral coordinators should also be applied to empower the integration.^18^^,^^32^^,^^33^

### To improve physical access of oral health services for older adults

Older adults who live in remote areas from oral health care services, have physical disabilities, or are institutionalized have difficulties seeking oral health care.^34^ As people age, they may have a higher chance of being institutionalized or dependent. Three quarters of older adults in India reside in rural areas and are required to travel a long way for oral health care due to the low dentist to population ratio.^19^ Almost 4 million older adults in Japan lived in long-term facilities and required escort or transportation for dental access.^35^ These physical barriers refrain older adults from having regular dental visits to maintain their oral health. Older adults may seek oral care at a later stage of oral diseases until they are in unbearable pain. Teledentistry, mobile dental care, and subsidized transportation have been proposed to facilitate dental access for older adults.^11^

Teledentistry is used for providing oral health care services to vulnerable groups like older adults. It also facilitates dental access to those who live in remote areas or places with a shortage of oral health care professionals.^36^ Its importance was more apparent during the COVID pandemic when physical distancing was imposed in many Asia-Pacific regions.^36^ Japan, China, India and Australia have applied teledentistry in oral diagnosis, oral health education, and postoperative follow-up.^36^ However, teledentistry requires advanced technical support and may not be easily applied in some Asia-Pacific region. Substantial funding for a fast, stable, and reliable internet network, patient confidentiality protection policy, and internet usage training for older adults and their caregivers are needed for the development of teledentistry.^36^^,^^37^

Mobile dental services have been successfully implemented as a means of providing outreach oral health care to older adults who are home-bound, institutionalized, physically disabled, or with special needs.^38^^,^^39^ Dependent older adults who received regular services from mobile dental services showed improved oral health.^39^ Mobile dental services have been delivered in several models. These include (1) mobile dental vehicle model with a self-contained facility transported from one location to another, (2) portable equipment model with equipment transported to another out-of-office location, (3) fixed equipment model with a dental clinic set up at the designated location and (4) the hybrid model.^39^ The type of oral care ranges from basic to complex interventions. The provision of oral care depends on the available resources and medical condition of older adults.^39^ Huge set up and maintenance costs for mobile dental vehicles, inadequate training in geriatric oral care, insufficient equipment and lack of collaboration among professionals are the barriers for mobile dental services.^39^^,^^40^ Therefore, the success of mobile dental services is much relied on the support and collaboration among government, nongovernmental organizations and all professional bodies.^38^ Mobile dental van services have been utilized as a means for postgraduate training in geriatric dentistry in India.^40^ It can increase the workforce in delivering oral health care to older adults and provide training in geriatric oral care to oral health care professionals.^19^^,^^40^

## Recommendations for international and national dental associations

### To conduct interprofessional training in geriatric oral care

Lack of experts in geriatric dentistry is one of the main obstacles to implementing oral health care policy in older adults in many Asia-Pacific regions.^19^ Lack of knowledge, skills and training also forbade other health care professionals in delivering oral health information and care.^32^ Dependent older adults relied on their care givers to maintain their proper oral hygiene. In Singapore, a study found that care givers in nursing homes demonstrated a good attitude towards the importance of oral health, however, half of them were not confident in providing oral health care without harming the patients.^41^ International and national dental associations bear the responsibilities in conducting lectures and workshop to strengthen the knowledge and skills of oral and other health-related professionals in geriatric oral care.

### To provide guidelines on the management of oral diseases in older adults

Traditional disease-centred intervention-oriented management approaches in dentistry could not reduce the burden of oral diseases especially in underprivileged groups like older adults.^42^ The intervention was usually costly and depended on high technology.^42^ This approach widened the inequalities in oral health and did not address the fundamental etiological factors of oral diseases.^42^ The international and national dental associations should emphasize the importance of preventive measures such as risk assessment, dietary advice, tobacco cessation and oral hygiene instruction and advocate the adoption of a patient-centred prevention-oriented management approach.^42^ The associations should formulate evidence-based clinical guidelines on the management of oral diseases in older adults for individual oral health care professionals.^14^ The Japanese Society of Conservative Dentistry has developed an evidence-based clinical guidelines for caries management using minimal intervention in adults.^43^ The Chinese Stomatological Association has recently issued the guidelines for prosthodontic treatment in older adults.^44^ The dental associations in other Asia-Pacific region should also develop guidelines for the management of oral diseases in older adults according to their sociodemographic background and risk factors.

### To advocate early detection of oral diseases

Older adults usually have their oral diseases treated at a later stage due to the physical and financial barriers to dental access. Delayed management may lead to more extensive damage and poorer clinical outcomes.^14^ Dental caries, if diagnosed at an earlier stage, may be possibly arrested and avoid any further invasive treatment. Oral function decline, if detected early, may be recovered by various orofacial muscular exercises before any irreversible dysfunction happens.^45^ International and national dental associations should stress the importance of early detection of oral diseases to the public and all health care professionals. The associations should advocate for individual oral health care professionals to include screening for oral cancer, hyposalivation and early signs of oral function decline as part of the routine dental checkup for older adults.^11^ It not only can manage oral diseases and oral function decline at the early stage to avoid extensive and complicated interventions, but also to minimize damage, improve clinical outcomes and enhance quality of life of the older adults.

### To conduct community-based oral health promotion and disease prevention programs

Preventive measures delivered in a downstream approach in individual clinical settings are far less effective than those given in an upstream population-wide approach.^42^ International and national dental associations should conduct more community-based oral health promotion and disease prevention programs to educate the public about the importance of oral health to general health for older adults, the need for regular dental checkup in detecting oral diseases in early stage and the merits of preventive measures of oral diseases. They can also co-organize with other health professional organizations to address the common risk factors in oral and systemic diseases of older adults. In Europe, the European Federation of Periodontology co-organized ‘The Perio and Cardio Project’ with the World Heart Federation to raise awareness of the links between periodontal diseases and cardiovascular diseases and the role of patients in the prevention of both diseases.^46^ Similar activities should be conducted in the Asia-Pacific region for older adults.

### To collect data for oral health status of older adults regularly as global surveillance

Regular data collection of the oral health status of older adults is imperative for formulating evidence-based cost-effective oral health policies.^47^ Data collection enables monitoring the oral health status of older adults and evaluating various interventions and oral health policies in terms of clinical outcome, economic impacts and implementation efficiency.^42^ The WHO advised to have clinical oral health data collected every 5 to 6 years for effective surveillance on disease patterns and trends.^48^ Data collection should be recorded in an integrated surveillance system using common oral health indicators.^42^ This integrated surveillance system should enable access by oral and other health care professionals to collect data for oral and systemic health of older adults.^18^ This allows comprehensive monitoring of the health status of older adults. The associations bear the responsibility to develop common oral health indicators and conduct data collection regularly for older adults. The WHO’s *Global oral health status report 2022* included the oral health profiles of countries in the Western Pacific Region based on data from the Global Burden of Disease to provide an estimation of the oral disease burden and highlight the challenges for universal coverage for oral health in these countries.^49^ Necessary data collection should be done for older adults in the Asia-Pacific region to aid oral health policy planning.

## Recommendations for academia

### To incorporate geriatric dentistry in university curriculum

Lack of experts in geriatric dentistry, an insufficient workforce and inadequate training in geriatric oral health care among health care professionals pose challenges in delivering oral health care to older adults and implementing oral health care policies.^19^^,^^50^ Incorporation of geriatric dentistry into the undergraduate curriculum is essential to train competent oral health care professionals to deliver oral health care for the increasing ageing population.^14^^,^^50^ Moreover, geriatric oral care should be included in undergraduate curriculum of other health-related schools such as social sciences, nursing and medicine.^14^ This can increase the potential workforce in providing oral health care for older adults and facilitate the integration of oral health into general health care.^14^ The curriculum should be designed with balanced didactic and clinical exposure to equip undergraduate students with basic knowledge such as common dental and medical problems in older adults and their interrelationship and clinical skills such as how to deliver oral health care for older adults with different needs and assisted oral hygiene practice.^14^^,^^50^ However, geriatric dentistry is currently not included in most of the dental schools in the Asia-Pacific regions.^19^

### To conduct research and provide evidence on improving oral health of older adults

Although population ageing has been a public health issue in many countries for years, research conducted about oral health care in older adult groups is scarce.^6^^,^^51^ Most of the clinical trials about oral health were conducted in children and adolescents and adult groups.^51^ The evidence on oral health care in older adults is lacking. Academia should assist with data collection for global surveillance on oral health conditions of older adults.^42^ They should conduct research to evaluate the effectiveness of various oral health care policies to provide feedback for policymakers to improve implementation.^14^ They should also assess different preventive measures and interventions on the management of oral diseases in older adults to provide evidence for oral and health care professionals in clinical practice.^42^

## Recommendations for individual oral health care professionals

### To manage oral problems using minimal invasive intervention at the early stage

Oral health care professionals should perform comprehensive examinations for all attending older adults to detect oral disease at an early stage. The examinations should include screening for oral cancer and assessment of oral function for older adults.^11^ The Japanese Society of Gerodontology has proposed a checklist of seven aspects including oral hygiene, oral dryness, occlusal force, tongue-lip motor function, tongue pressure, masticatory function and swallowing function for the assessment of oral hypofunction.^45^ Furthermore, oral health care professionals should prioritize preventive measures and apply minimally invasive approach for the management of oral diseases in older adults.^52^ Fluoride therapy is well known for caries management.^53^ Self-applied high dose fluoride toothpaste and professionally applied silver diamine fluoride solution have shown effectiveness in arresting root caries in older adults.^51^^,^^54^ They are inexpensive, noninvasive and simple to use and hence should be considered as the first line for caries management in older adults.^51^

### To collaborate with other health care professionals

Some systemic diseases may present early signs in the form of ulcers, white or red lesions, halitosis, dry mouth, or bleeding gums in the oral cavity.^55^ Oral health care professionals should be aware of these early signs of systemic diseases and refer the older adults for further medical consultation, if indicated. They should utilize the common risk factors approach to promote the oral and general health of older adults to improve their overall well-being.^24^^,^^42^ Dietary advice should not only focus on preventing oral diseases but also avoiding overweight or malnutrition in older adults.^18^ Oral health care professionals should encourage tobacco cessation using 5A model through ask, advise, assess, assist and arrange.^56^ They should arrange for behavioural counselling or proper referrals to the national smoking cessation units, if needed.^56^ Besides smoking tobacco, betel quid chewing is also a risk factor for lip and oral cavity cancer and is widespread in South-East Asia and the Pacific Islands.^57^ Older adults in the Asia-Pacific region should be educated about its association with oral cancer and be encouraged to cease betel quid chewing. Oral health care professionals should always be equipped to provide advice or a workforce for the health care system to relieve the burden of the increasing ageing population.

The above recommendations provided some insights on how to redesign the oral health care policies and guidelines in oral health care for older adults. However, there will be region-specific differences, and the recommendations may not be fitted in for all Asia-Pacific regions. Modifications may be warranted based on regions’ circumstances. Table summarizes the recommendations for different stakeholders in improving the oral health care of older adults.

**Table:** The recommendations for different stakeholders in improving the oral health of older adults.

**Actions (*stakeholders*)**	**Rationale**
***Policymakers***	
*To include oral health in universal health coverage*	Reducing oral health inequalities, and make oral health care more accessible, available, and affordable to older adults
*To integrate oral health into general health care system*	Providing a patient-centred, integrated and multidisciplinary approach to improve oral and systemic health of older adults
*To improve physical access of oral health services for older adults*	Lowering physical barrier to oral health care for older adults in remote areas, in long-term care facilities, or with special needs
***International and national dental associations***	
*To conduct interprofessional training in geriatric oral care*	Equipping oral and other health care professionals in delivering geriatric oral care, and facilitate the integration of oral and general health care services in older adults
*To provide guidelines on the management of oral diseases in older adults*	Advocating patient-centred, prevention-oriented approach in managing oral diseases in older adults
*To advocate early detection of oral diseases*	Avoiding cost, extensive and complicated treatment, and to improve clinical outcomes in older adults
*To conduct community-based oral health promotion and disease prevention programs*	Adopting population-wide upstream approach in tackling oral diseases in older adults
*To collect data for oral health status of older adults regularly as global surveillance*	Providing evidence to guide and support oral health policy
***Academia***	
*To incorporate geriatric dentistry in university curriculum*	Equipping future oral and other health care professionals with knowledge and skills in delivering geriatric oral care
*To conduct research and provide evidence on improving oral health of older adults*	Providing evidence for the best practice on improving oral health in older adults
***Individual oral health care professionals***	
*To manage oral problems using minimal invasive intervention at the early stage*	Avoiding cost, extensive and complicated treatment, and to improve clinical outcomes in older adults
*To collaborate with other health care professionals*	Advocating patient-centred prevention-oriented approach in managing oral diseases in older adults

## Conclusions

The population is ageing, and oral disease is prevalent among older adults in the Asia-Pacific region. Oral health is crucial for healthy ageing. All stakeholders are responsible for improving oral health for older adults in the Asia-Pacific region. The recommendations provide some insights on how to improve the oral health care policies, health care system, and oral health care promotion and practice for older adults.

## CRediT authorship contribution statement

**Alice Kit Ying Chan:** Writing – original draft, Methodology. **Chun-Pin Lin:** Writing – review & editing, Funding acquisition. **Olabode Ijarogbe:** Resources, Validation. **Elham Kateeb:** Resources, Validation. **Hiroshi Ogawa:** Resources, Validation. **Syed Mahmood Shah:** Resources, Validation. **Fernando Fernandez:** Resources, Validation. **Ting-Chen Chen:** Resources, Validation. **Ting-Yi Renn:** Writing – review & editing, Validation. **Chun Hung Chu:** Conceptualization, Writing – review & editing, Supervision. **Wei-Jen Chang:** Conceptualization, Writing – review & editing, Supervision.

## Conflict of interest

The authors declare that they have no known competing financial interests or personal relationships that could have appeared to influence the work reported in this article.
